# Trephining for Depressed Fracture in Vertex of Skull

**Published:** 1883-02

**Authors:** Parker Syms

**Affiliations:** House Surgeon, Bellevue Hospital


ARTICLE II.

### TREPHINING FOR DEPRESSED FRACTURE IN VERTEX OF SKULL.

BY PARKER SYMS, M. D.,
House Surgeon, Bellevue Hospital.

A laborer, aged thirty-six, was brought to one of my wards on the afternoon of November 1, 1882, suffering from a compound depressed fracture of the skull.

He stated that a few hours before his admission to the hospital he had been struck on the top of the head by a stone.

His pupils were equal in size, rather dilated, and both responded to the action of light. His intellect was good, and he showed no symptoms of cerebral compression, except a slow, full pulse (between fifty and sixty), and slight paresis of both lower extremities.

Examination showed a not very large scalp wound in the vertex, almost directly in the median line.

On enlarging this wound, a small piece of the outer table of the skull was found to be depressed.

It was decided to trephine and elevate the depressed bone.

The patient was etherized, and the scalp laid further back by making crucial incisions.

When the bone was brought more clearly to view, I found that the fracture through the outer table was limited to a small fragment, about half an inch in length and a quarter of an inch in width. All of this was considerably depressed.

This depressed fragment was directly over the superior longitudinal sinus, its long diameter corresponding to the transverse diameter of the skull.

Using a small trephine, a button of bone was removed from the skull, just to the left of the fragment. The fragment was very easily dislodged with an elevator. By using a probe, it was found that the inner table was fractured in all directions.

In order to remove the loose pieces without injuring the dura, I cut away the margin of the opening I had already made, with a pair of rongeur forceps. I removed at least a dozen pieces of bone, which had been fractured off from the inner table and lodged between the bone and dura mater.

To remove all of these, it was necessary to enlarge the opening, making it at least two inches in diameter.

After removing all the loose fragments of bone, I smoothed off the edges of the opening and packed the wound with picked lint soaked in carbolized oil.

By the third day the wound had begun to granulate from the bottom. It went on, without complication, to a complete cure.

The patient slowly recovered from his paresis, and now has perfect use of his limbs.

This case seems to me to be of special interest, as showing the necessity of trephining in depressed fracture of the vertex, even though there be no urgent symptoms; for any one of these pieces, fractured from the inner table, would probably have caused a fatal meningitis, had I not removed them.

